# Retinal microvascular changes in patients with familial mediterranean fever: a study based on optical coherence tomography angiography

**DOI:** 10.5935/0004-2749.20220038

**Published:** 2025-08-21

**Authors:** Seyfettin Erdem, Mine Karahan, Sedat Ava, Mehmet Emin Dursun, Figen Ceylan Cevik, Emin Özkul, Ugur Keklikci, Remzi Çevik

**Affiliations:** 1 Department Ophthalmology, Dicle University Medical Faculty, Diyarbakır, Turkey; 2 Department of Physical Medicine and Rehabilitation, Fizyopolitan PMR Center, Diyarbakır, Turkey; 3 Department of Orthopaedics and Traumatology, Dicle University Medical Faculty, Diyarbakır, Turkey; 4 Department of Physical Medicine and Rehabilitation, Division Rheumatology, Dicle University Medical Faculty, Diyarbakır, Turkey

**Keywords:** Optical coherence tomography angiography, Familial Mediterranean fever, Retinal microcirculation, Superficial plexus, Deep vascular capillary plexus, Tomografia de coerência óptica, Microcirculação retiniana, Febre mediterrânea familiar, Plexo superficial, Densidade dos vasos do plexo capilar

## Abstract

**Purpose:**

In this study, we aimed to show whether a difference exists between retinal and choroidal microcirculation findings between patients with familial Mediterranean fever and healthy controls.

**Methods:**

Thirty-two patients with familial Mediterranean fever and 30 healthy controls were included in the study. All the patients underwent a complete ophthalmologic examination, including best-corrected visual acuity and intraocular pressure measurement. The AngioVue optical coherence tomography angiography device (Optovue, Fremont, CA) with split-spectrum amplitude-decorrelation angiography was used to evaluate and examine the retinal microvascular structure. Three-dimensional en face Optical coherence tomography angiography images were obtained by examining the macula using the 3 x 3 mm scanning protocol in the Angio Retina mode and the optic nerve using the 3 x 3 mm scanning protocol in the Angio Disk mode. All the patients’ right eyes were examined.

**Results:**

A total of 62 subjects were included in the study, of whom 32 (53.3%) were female and 30 (46.7%) were male. No statistically significant difference was found between the two groups in terms of optic nerve head or radial peripapillary capillary vessel density. On examination, the superficial capillary plexuses were statistically similar between the two groups, but the deep capillary plexus vessel density in the parafovea, superior hemi, temporal, and superior areas were significantly lower in the patients with familial Mediterranean fever.

**Conclusions:**

We found that the capillary plexus vessel density was significantly lower in the parafovea, superior hemi, temporal, and superior regions in the patients with familial Mediterranean fever than in the control group. Therefore, OCTA, a noninvasive study, may be useful for understanding the systemic effects of familial Mediterranean fever.

## Abstract (pt)

**Objetivos:**

Este estudo teve como objetivo mostrar se há diferença entre os achados da microcirculação retiniana e coroidal entre pacientes com febre mediterrânica familiar e um grupo controle saudável.

**Métodos:**

Trinta e dois pacientes com febre mediterrânica familiar e 30 controles saudáveis foram incluídos neste estudo. Todos os pacientes foram submetidos a um exame oftalmológico completo, incluindo a acuidade visual melhor corrigida e medida da pressão intraocular. O aparelho AngioVue Optical coherence tomography angiography (Optovue, Fremont, CA) com angiografia de correlação de amplitude de espectro dividido foi utilizado para avaliar e examinar a estrutura microvascular da retina. As angiotomografias de coerência ópticas *en face* tridimensionais foram obtidas examinando o protocolo de varredura macular 3 x 3 mm (modo angio retina) e o nervo óptico com o protocolo de varredura 3 x 3 mm (modo angio-disco). Todos os olhos direitos dos pacientes foram examinados.

**Resultados:**

Foram incluídos neste estudo, 62 sujeitos, dos quais 32 (53,3%) eram do sexo feminino e 30 (46,7%) do sexo masculino. Não houve diferença estatisticamente significativa entre os dois grupos quanto à densidade dos vasos da cabeça do nervo óptico ou da densidade dos vasos capilares peripapilares radiais. Durante o exame, os plexos capilares superficiais foram estatisticamente semelhantes entre esses dois grupos, mas a densidade profunda dos vasos do plexo capilar nas areas parafovea, hemi superior, temporal e superior foram significativamente menores nos pacientes com febre mediterrânica familiar.

**Conclusões:**

Verificamos que a densidade dos vasos do plexo capilar foi significativamente menor nas regiões parafovea, hemi superior, temporal e superior em pacientes com febre mediterrânica familiar em comparação com o grupo controle. Portanto, pode ser útil usar a angiotomografia de coerência óptica, por tratar-se de um estudo não invasivo, para melhor compreensão dos efeitos sistêmicos da febre mediterrânica familiar.

## INTRODUCTION

Familial Mediterranean fever (FMF), the most common autoinflammatory disorder, has an autosomal recessive pattern of inheritance, and depending on the geographic region, the prevalence of the disease ranges from 1 per 250 population to 1 per 1000 population. This disease has been reported mostly from the Middle East and Mediterranean countries, and to a lesser extent from places such as European countries, the United States, and Japan^(1-3)^.

In FMF, the role of the prin protein in the regulation of natural immunity is inhibited owing to missense mutations in the Mediterranean fever (*MEFV*) gene, which changes the structure and function of the pyrin protein^(4)^. Forms of serositis, such as peritonitis, pleuritis, and arthritis, occur because of prolonged and increased inflammation, which are accompanied by fever in patients with FMF^(5,6)^. During these attacks, high erythrocyte sedimentation rate, neutrophilic leukocytosis, and increased fibrinogen, C-reactive protein, and serum amyloid A (SAA) levels are observed^(7)^. Increased incidence rates of diseases such as spondyloarthritis, multiple sclerosis, ulcerative colitis, and vasculitis such as IgA vasculitis and polyarthritis nodosa (PAN) have also been reported in FMF^(8,9)^.

In previous studies, mostly case reports, that examined eye findings in patients with FMF, ocular findings such as uveitis, retinal diseases, amaurosis fugax, optic neuritis, and ocular surface and tear film abnormalities have been reported during attacks in the patients with FMF^(10-13)^. However, when the literature is examined, a limited number of studies were on retinal and choroidal vascular changes in patients with FMF^(14)^.

The increased inflammation occurring with the disease may make the eye tissues, where vascular structures are concentrated, like other systems, sensitive to the effects of inflammatory and vascular systemic diseases. Therefore, changes in the retinal and choroidal vascular structures may be caused by the vasculopathy and inflammatory nature of the disease. The large vessels in the eye are in the outermost layer of the choroid, while the small ones are in the choriocapillaris and retina.

Optical coherence tomography angiography (OCTA), which is a noninvasive, fast, safe, and reproducible imaging method, provides high-resolution visualization of the retinal tissue and measures the dimensions of retinal capillary networks and foveal avascular zones (FAZ)^(15-17)^. An analysis of retinal microcirculation networks such as vascular density (VD) of the retinal capillary plexuses, optical disk head, radial peripapillary capillary (RPC-VD), and FAZ has not been performed with OCTA in patients with FMF.

In this study, we aimed to show whether a difference exists in retinal and choroidal microcirculation findings obtained using OCTA, a noninvasive method, between patients with FMF and healthy controls.

## METHODS

### Study design and subjects

Between January 2020 and March 2020, 32 patients with FMF and 30 healthy controls were included in the study. Approval was obtained from the ethics committee of Dicle University School of Medicine. Our study was conducted in accordance with the Declaration of Helsinki, and written informed consent was obtained from all the patients before the measurement.

In this cross-sectional study, patients with chronic diseases such as diabetes mellitus and hypertension, neurological diseases, collagen tissue diseases, and ocular diseases such as previous intraocular surgery, ca taract, history of ocular trauma, history of glaucoma, corneal opacity, and retinal disease and those who did not cooperate for OCTA screening were excluded. The patients with FMF were evaluated by the Division of Rheumatology, Department of Physical Medicine and Rehabilitation, Dicle University Hospital, and referred to the Department of Ophthalmology for eye examination. All the patients with FMF fulfilled the Tel Hashomer diagnostic criteria^(18)^.

Assessment of disease severity was evaluated using the scoring system of Pras et al.^(19)^. The scoring system has six elements, including age of onset, colchicine dose, number of attacks per month, presence of arthritis, erysipelas-like erythema, and amyloidosis. According to their scores, the patients were classified into three groups as follows: mild (2-5 points), moderate (6-10 points), and severe (>10 points).

The mean age of the control group was similar to that of the FMF group. A complete ophthalmological examination was performed in all the patients, including best-corrected visual acuity, intraocular pressure measurement, and slit-lamp biomicroscopy.

### Optical coherence tomography angiography measurements

In our study, the AngioVue OCTA device (Optovue, Fremont, CA) was used to obtain split-spectrum amplitude-decorrelation angiograms (version 2016.2.0.35). An A-scan image was obtained with a light source centered at 840 nm with a scanning speed of 70,000/s and a bandwidth of 50 nm. Three-dimensional en face OCTA images were obtained by examining the macula using the 3 x 3 mm scanning protocol in the Angio Retina mode and the optic nerve using the 3 x 3 mm scanning protocol in the Angio Disk mode. All the patients’ right eyes were examined.

**Figure 1:**
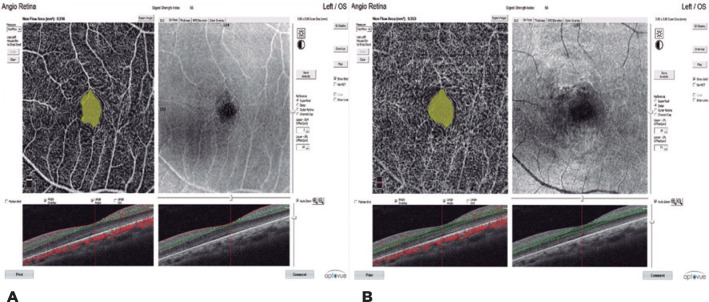
A) Superficial FAZ. B) Deep FAZ.

**Figure 2:**
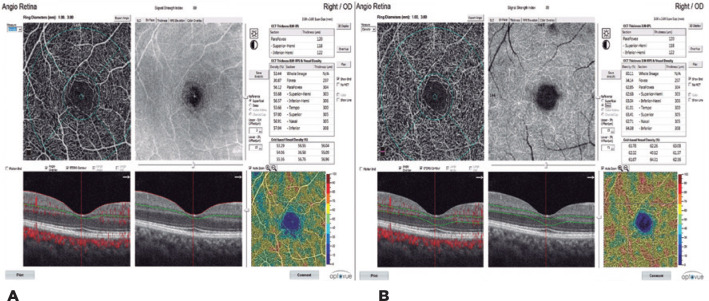
A) Superficial capillary plexus vascular density (SCP-VD). B) Deep capillary plexus vascular density (DCP-VD).

**Figure 3:**
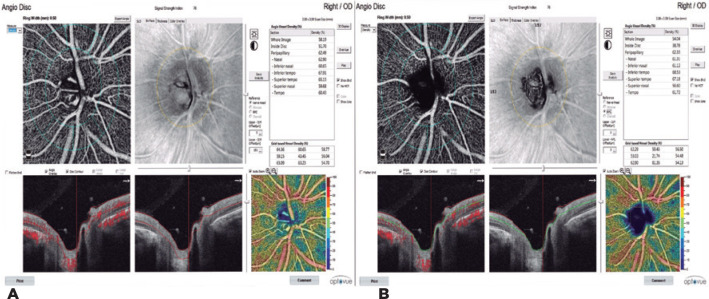
A) Optic nerve head (ONH-VD). B) Radial peripapillary capillary density (RPC-VD).

The non-flow assessment tool in the OCTA software version was used to calculate the FAZ areas in the superficial capillary plexus (SCP) and deep vascular capillary plexus (DCP; Figure 1 A, B), while the VD was calculated as the percentage area occupied by the blood vessels. By performing superficial and deep macular scans, the VDs of the fovea, parafovea (the region between the outside diameter of 3 mm and the inside diameter of 1 mm; temporal, superior, nasal, and inferior), superior hemi, and inferior hemi areas were calculated in both the SCP (SCP-VD) and DCP (DCP-VD; Figure 2 A, B). The VDs of the optic nerve head (ONH) and RPC network were measured using ONH scanning. With this scan, both RPC-VD and ONH-VD were calculated from six regions (nasal, inferonasal, inferotemporal, superotemporal, superonasal, and temporal). The region extending from the optic nerve border as an ellipse-shaped ring with a width of 0.75 mm was defined as the peripapillary area (Figure 3 A, B).

### Statistical analyses

We performed all statistical analyses using the SPSS version 26.0 software (SPSS Inc., Chicago, IL, USA). Demographic data were calculated using descriptive statistics. The mean and standard deviations were used to describe the data. The Kolmogorov-Smirnov test was used to assume a normal distribution of the variables, and an independent *t* test and chi-square test were used to compare continuous variables.

## RESULTS

A total of 62 subjects were included in the study, of whom 32 (53.3%) were female and 30 (46.7%) were male. The mean age of the subjects was similar in both groups (59.80 ± 6.16 and 51.50 ± 5.83 years, respectively). All the patients included in the study were receiving treatment (colchicine, 100% and biologic agent, 9.4%). The mean disease duration was 15.00 ± 7.69 years (Table 1). The *MEFV* gene mutations in the patients with FMF are summarized in table 2. In this study, *M694V* mutations were the most common in the *MEFV* genetic analysis. The comparison of the FMF and control groups revealed no statistically significant difference between the groups in terms of ONH-VD or RPC-VD (Table 3). While all the SCP parameters were statistically similar between the two groups, upon examination, the DCP-VDs in the parafovea, superior hemi, temporal and superior regions were significantly lower in the FMF group than in the control group (Table 4). In addition, although both the superficial and deep FAZs were larger in the patients with FMF, this difference was not statistically significant (Table 5).

**Table 1:** Demographic characteristics of the patients included in the study

Characteristic	Patients with FMF (n=32)	Control (n=30)	Significant (p value)
Age (years), mean ± SD	30.65 ± 8.64	34.10 ± 6.84	0.089
Sex, n (%) Female (30, 48.4) Male (32,51.6)	16(50.0) 16(50.0)	14 (46.7) 16(53.3)	0.797
VAS score	7.24 ± 1.99	-	-
Disease severity score	6.07 ± 2.54	-	-
Medication (%)	Colchicine (100) Biologic agent (9.4)		
Disease duration (years)	15.00 ± 7.69	-	-
Diagnostic delay (years)	6.41 ± 7.73		

**Table 2:** *MEFV* gene mutations in the patients with FMF

MEFV mutation	Homozygous n (%)	Heterozygous n (%)	Compound heterozygous n (%)
M694V	4(12,5)	7(21,9)	5 (15,6)
V726A	1 (3,12)	4(12,5)	1 (3,12)
M6801	3 (9,37)	-	-
E148Q	-	2 (6,25)	1 (3,12)
R202Q	-	2 (6,25)	2 (6,25)

**Table 3:** Optic nerve head (ONH-VD) and radial peripapillary capillary vascular densities (RPC-VD) of the patients included in the study (%)

Characteristic	ONH-VD^*^			RPC-VD^**^		
	Patients with FMF	Control	Significant *(p)*	Patients with FMF	Control	Significant *(p)*
Whole Image	61.00 ± 2.16	60.43 ± 3.15	0.41	59.34 ± 2.35	58.83 ± 2.98	0.45
Inside disk	57.76 ± 4.08	56.05 ± 5.31	0.15	50.33 ± 9.34	50.04 ± 7.54	0.89
Peripapillary	63.30 ± 2.42	63.68 ± 2.98	0.57	65.08 ± 3.12	65.17 ± 3.35	0.90
Nasal	61.93 ± 2.89	62.50 ± 4.02	0.52	62.72 ± 3.46	63.35 ± 5.24	0.57
Inferonasal	64.26 ± 4.43	64.70 ± 5.00	0.71	65.89 ± 4.72	65.63 ± 5.67	0.84
Inferotemporal	66.64 ± 4.51	65.96 ± 4.44	0.55	69.48 ± 5.17	68.82 ± 4.50	0.59
Superotemporal	63.75 ± 4.66	65.00 ± 4.33	0.28	67.46 ± 4.52	68.33 ± 4.80	0.46
Superonasal	62.07 ± 4.87	63.64 ± 4.96	0.21	62.22 ± 5.97	64.07 ± 5.28	0.20
Temporal	63.47 ± 3.41	62.95 ± 4.80	0.62	65.75 ± 4.17	64.44 ± 4.75	0.25

* = Optic nerve head (ONH-VD) and

** = radial peripapillary capillary density (RPC-VD) of the patients included in the study (%).

**Table 4:** Superficial capillary plexus (SCP-VD) and deep capillary plexus vascular densities (DCP-VD) of the patients included in the study (%)

Characteristic	SCP-VD^*^			DCP-VD^**^		
	Patients with FMF	Control	Significant *(*p* value)*	Patients with FMF	Control	Significant *(*p* value)*
Whole Image	53.81 ± 2.22	53.99 ± 1.75	0.71	60.32 ± 1.51	60.88 ± 1.63	0.16
Fovea	31.99 ± 6.24	30.05 ± 5.21	0.19	32.94 ± 6.66	29.86 ± 6.38	0.07
Parafovea	56.04 ± 2.71	56.38 ± 1.78	0.56	62.90 ± 1.71	63.79 ± 1.70	0.04^*^
Superior hemi	55.97 ± 2.67	56.33 ± 1.51	0.51	62.81 ± 1.72	63.88 ± 1.70	0.02^*^
Inferior hemi	56.11 ± 2.85	56.43 ± 2.20	0.62	62.99 ± 2.04	63.68 ± 1.86	0.16
Temporal	54.65 ± 2.75	55.18 ± 1.95	0.39	61.63 ± 2.03	62.68 ± 1.77	0.03^*^
Superior	56.82 ± 2.95	56.79 ± 2.39	0.96	63.77 ± 1.66	64.94 ± 1.96	0.01^*^
Nasal	55.79 ± 2.71	55.87 ± 2.13	0.90	62.33 ± 1.84	63.17 ± 2.12	0.10
Inferior	56.94 ± 3.20	57.42 ± 2.31	0.49	63.87 ± 2.31	64.37 ± 2.01	0.36

SCP-VD= superficial capillary plexus vessel density; DCP-VD= deep capillary plexus vessel density.

* *p<*0.05.

**Table 5:** Foveal avascular zones of the patients included in the study (%)

FAZ (mm^^2^^)	Patients with FMF	Control	Significant *(p)*
Superficial	0.308 ± 0.88	0.281 ± 0.10	0.28
Deep	0.336 ± 0.09	0.317 ± 0.09	0.42

## DISCUSSION

In this study, we aimed to reveal whether a difference exists in retinal microcirculation findings between patients with FMF and healthy controls by using a noninvasive OCTA method. As a result, we found that the DCP-VDs in the parafovea, superior hemi, temporal, and superior regions were significantly lower in the FMF group than in the control group. Similarly, the deep FAZ area was statistically significantly larger in the FMF group.

In FMF, apoptosis causes the release of caspase-1 enzyme interleukin (IL) 1, which is activated by mutations in the *MEFV* gene that encodes pyrin, which is responsible for the inflammation and regulation of cytokines. The released IL-1β also leads to the activation and production of tumor necrosis factor-alpha (TNFα)^(20,21)^. The cause of inflammation in the patients with FMF is these proinflammatory cytokines. Serum IL-1β and TNFα levels have been shown to be high in patients with FMF both during acute attacks and during non-attack periods^(22,23)^.

Studies have reported the effects of cytokines such as IL-1β and TNFα on retinal structures. Moreover, these cytokines have been reported to induce optic neuropathy and retinal ganglion cell degeneration in animal studies. Serum TNFα levels have been reported to be high in diabetic patients with diabetic retinopathy (DR), a microangiopathic complication of diabetes^(24)^. The increase in the levels of these cytokines both in the patients with FMF and those with DR may explain the decrease in the VD of the retinal deep capillary plexus that we detected in the patients with FMF who had similar physiopathological mechanisms.

The risk of chronic inflammation has been reported to increase the risk of endothelial dysfunction and atherosclerosis, even during remission, and vascular diseases such as coronary artery disease and pulmonary hypertension can be observed^(25,26)^. Therefore, revealing possible changes in retinal and choroidal microvascular structures in these patients may contribute to further elucidation of the pathophysiology of the disease.

We found that the DCP-VDs in the parafovea, superior hemi, temporal, and superior regions were low in the patients with FMF. Similarly to our study, deep inferior and deep inferior hemi VDs have been reported to be significantly decreased in patients with FMF than in healthy controls^(14)^. These results suggest that deep retinal microvascular structures may be more susceptible to inflammation. In addition, a negative correlation was found between the temporal quadrant retinal nerve fiber layer (RNFL) thickness and disease duration in a study that used OCT to investigate the effect of inflammation in patients with ankylosing spondylitis, an autoinflammatory disease^(27)^. These results suggest that the microvascular structures of the temporal quadrant may be more susceptible to inflammation. On the other hand, in several studies that used OCT, the peripapillary RNFL and retinal GCIPL thickness of patients with FMF were reported to be similar to those of controls^(28)^. Similarly, the retinal and choroidal thicknesses were reported to be similar between children with FMF in remission and controls^(29)^.

In our study, in accordance with the literature, we found deep FAZ changes in the patients with FMF. Increased FAZ, which may be an indication of decreased foveal microcirculation and macular ischemia, has been previously reported in other diseases^(30,31)^.

In the present study disease severity was evaluated and found to be moderate according to the scoring system of Pras et al.^(19)^ All the patients received treatment with colchicine and biologic agents, and none had an acute attack. However, moderate disease severity and high diagnostic delay may have led to the microvascular changes observed in our patients. Therefore, in patients with acute attack or high severity score, microvascular structures in other regions of the deep vascular complex and other parts of the retina may also be affected.

Our study has some limitations. First, the results of a single-center study cannot be generalized for all patients with FMF. Second, the number of samples included in our study was small. Third, long-term follow-up of the patients was lacking. Fourth, none of the patients with an acute attack was included in the study. However, FMF attacks have the potential to cause some changes in the retinal vascular structures due to the proinflammatory nature of the disease. The strength of this study is that it can contribute important information in the literature, as the number of studies on this subject are limited.

In conclusion, we found that DCP-VD was significantly lower in the parafovea, superior hemi, temporal, and superior regions in the patients with FMF than in the controls. Moreover, the deep FAZ area was found to be larger in the patients with FMF. Therefore, especially in patients with FMF, the use of OCTA, a noninvasive and easily applicable method, can be useful for both understanding the systemic effects of the disease and the possible pathophysiological mechanisms of the disease by evaluating the potential risk of possible microvascular complications. However, studies with multicenter and large patient series may contribute to the literature on this subject in the future.
